# New Kids on the Block: Estimating Use of Next-generation Gram-negative Antibiotics Across Greater Than 700 Hospitals in the United States

**DOI:** 10.1093/ofid/ofaf079

**Published:** 2025-02-12

**Authors:** Anthony D Harris, Katherine E Goodman, Lisa Pineles, Morgan Walker, Jacqueline T Bork, Emily L Heil, Kimberly C Claeys, Justin Brooks, Sameer Kadri, Bradley A Maron, Jonathan D Baghdadi

**Affiliations:** Department of Epidemiology and Public Health, University of Maryland School of Medicine, Baltimore, Maryland, USA; UM Institute for Health Computing, North Bethesda, Maryland, USA; Department of Epidemiology and Public Health, University of Maryland School of Medicine, Baltimore, Maryland, USA; UM Institute for Health Computing, North Bethesda, Maryland, USA; Department of Epidemiology and Public Health, University of Maryland School of Medicine, Baltimore, Maryland, USA; UM Institute for Health Computing, North Bethesda, Maryland, USA; Critical Care Medicine Department, Clinical Center, National Institutes of Health, Bethesda, Maryland, USA; Department of Practice, Sciences, and Health Outcomes Research, University of Maryland School of Pharmacy, Baltimore, Maryland, USA; Department of Practice, Sciences, and Health Outcomes Research, University of Maryland School of Pharmacy, Baltimore, Maryland, USA; Department of Practice, Sciences, and Health Outcomes Research, University of Maryland School of Pharmacy, Baltimore, Maryland, USA; UM Institute for Health Computing, North Bethesda, Maryland, USA; Department of Computer Science and Electrical Engineering, University of Maryland Baltimore County, Catonsville, Maryland, USA; Critical Care Medicine Department, Clinical Center, National Institutes of Health, Bethesda, Maryland, USA; Department of Epidemiology and Public Health, University of Maryland School of Medicine, Baltimore, Maryland, USA; UM Institute for Health Computing, North Bethesda, Maryland, USA; Department of Epidemiology and Public Health, University of Maryland School of Medicine, Baltimore, Maryland, USA; UM Institute for Health Computing, North Bethesda, Maryland, USA

**Keywords:** gram-negative antibiotics, infections, new antibiotics, gram-negative bacteria, hospital discharge data

## Abstract

**Background:**

In recent years, new broad-spectrum antibiotics targeting Gram-negative organisms have been introduced, including cefiderocol, ceftazidime-avibactam, ceftolozane-tazobactam, eravacycline, imipenem-relebactam, omadacycline, and meropenem-vaborbactam. This study aimed to describe new antibiotic use across a large national cohort.

**Methods:**

We performed a retrospective cohort study of hospital discharges from June 2022 to May 2023 using the Premier Healthcare Database. Antibiotic utilization was ascertained from daily charges. Clinical indication(s) were inferred from International Classification of Diseases, 10th revision, diagnosis codes. Antibiotic therapy was considered definitive if continued >3 days. Piperacillin-tazobactam was used as a comparator.

**Results:**

Across 832 hospitals, 3 890 557 admissions (61.9% of all admissions) included an antibiotic prescription. New antibiotics were prescribed in 9768 admissions (0.25% of antibiotic-prescribing admissions) across 537 hospitals. Ceftolozane-tazobactam was prescribed in 4157 admissions (42.6% of 9768), ceftazidime-avibactam in 3660 (37.5%), eravacycline in 1213 (12.4%), cefiderocol in 1060 (10.9%), meropenem-vaborbactam in 456 (4.7%), omadacycline in 104 (1.1%), and imipenem-relebactam in 99 (1.0%). In contrast, piperacillin-tazobactam was prescribed in 731 719 (18.8%) and colistin in 570 (0.01%) admissions. Forty-six percent (n = 4647/9768) of new antibiotics were started in the first 3 days of hospital admission, and 70% (n = 6799/9768) were used as definitive therapy. Sepsis (76%), pneumonia (46%), and urinary tract infection (39%) were the most common clinical indications. On average, patients treated with new antibiotics had 8 more comorbid conditions than patients receiving piperacillin-tazobactam.

**Conclusions:**

Ceftazidime-avibactam and ceftolozane-tazobactam remain the most frequently prescribed new antibiotics, with uptake of subsequently approved agents trailing. New antibiotics are most commonly used as treatment for sepsis among patients with multiple comorbidities.

In the last decade, the U.S. Food and Drug Administration has approved multiple new antibiotics with broad-spectrum Gram-negative activity: cefiderocol, ceftazidime-avibactam, ceftolozane-tazobactam, eravacycline, imipenem-relebactam, omadacycline, and meropenem-vaborbactam. These antibiotics are particularly effective against important emerging Gram-negative pathogens such as carbapenem-resistant *Enterobacterales* (CRE).

Though evidence suggests that these new antibiotics are under-utilized [1], studies have not examined the clinical indications for which they were prescribed. Further, use of new Gram-negative antibiotics has not been described in the post-COVID era. The objectives of this study were: (1) to describe the use of new Gram-negative antibiotics in a recent time period (2022–2023), (2) to understand the clinical indications for which these antibiotics were used, and (3) to compare the characteristics of patients and infections treated with new antibiotics against piperacillin-tazobactam, a widely used older Gram-negative antibiotic.

## METHODS

We conducted a retrospective observational cohort study of adult patient hospitalizations between May 2022 and May 2023 from hospitals in the Premier Healthcare Database (also known as PINC AI). PINC-AI is an all-payer database encompassing greater than 120 million hospitalizations at approximately 25% of US hospitals. This dataset has been used previously to address infectious disease questions, including by the Centers for Disease Control and Prevention [2–9]. This study did not include protected health information and was deemed exempt human subjects research by the University of Maryland institutional review board.

### Cohort, Definitions and Collected Data

We created a cohort of patients treated with the following new antibiotics: cefiderocol, ceftazidime-avibactam, ceftolozane-tazobactam, eravacycline, imipenem-relebactam, omadacycline, and meropenem-vaborbactam. We then compared the demographics and clinical indications of this new antibiotic cohort with a second cohort consisting of patients who received piperacillin-tazobactam, which is a widely used Gram-negative antibiotic [10, 11].

For both cohorts, we extracted medication charge data and International Classification of Diseases, 10th revision, diagnosis codes. We mapped diagnosis codes to Elixhauser comorbidities using validated Agency for Healthcare Research and Quality methodology [7] and to 4 infectious syndromes selected for clinical relevance (pneumonia [12], urinary tract infection [13], abdominal infection [14], and sepsis [15–17]; code sets are listed in Supplementary Appendix 1). Infectious diagnoses were not mutually exclusive. Diagnosis of sepsis included explicit diagnosis of sepsis or implicit diagnosis based on presence of infection plus organ dysfunction. A stratified analysis was performed among patients who received a new antibiotic as definitive therapy, which was defined by treatment for >3 consecutive days. Comparisons among groups were made using the Fisher exact 2-tailed test for significance. All statistical tests were 2-tailed with a threshold of ≤0.05 for significance of *P* values. Statistical analyses were performed using SAS version 9.4 and StataMP 17 (StataCorp, College Station, Texas).

## RESULTS

**Figure 1.**
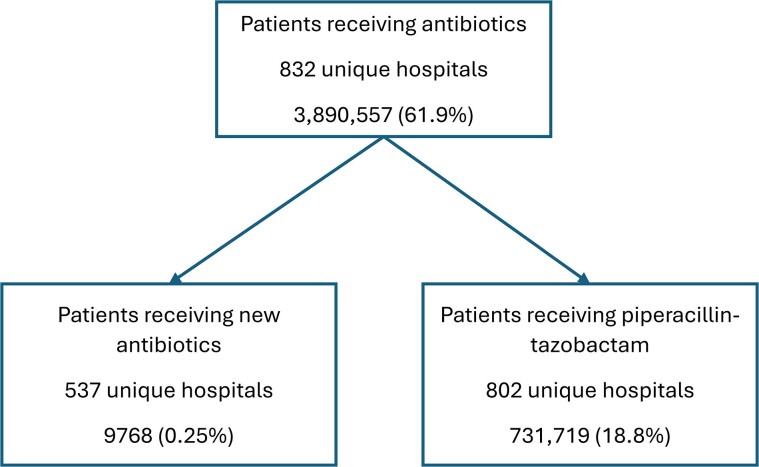
Antibiotic use among hospitals in study.

Across 832 hospitals, 3 890 557 admissions (61.9% of all admissions) were associated with antibiotic treatment (see Figure 1 for flow diagram). New antibiotics were prescribed in 9768 admissions (0.25% of antibiotic-prescribing admissions) across 537 hospitals. Piperacillin-tazobactam was prescribed in 731 719 admissions (18.8% of antibiotic-prescribing admissions), and colistin was prescribed in 570 admissions (0.01%).

Use of new antibiotics was clustered within a relatively small subset of hospitals. Ten hospitals accounted for 25% of admissions associated with new antibiotic use. Eight of these 10 hospitals were teaching hospitals (although the hospital with the largest volume of new antibiotic use was a nonteaching hospital). Fifty-one hospitals accounted for 50% of admissions associated with new antibiotic use. New antibiotics were not used at 294 hospitals (35.5% of hospitals associated with an antibiotic-prescribing admission).

Table 1 shows the prevalence of use of each new antibiotic. Ceftolozane/tazobactam was prescribed most frequently (42.6% of new antibiotic-prescribing admissions), followed by ceftazidime avibactam (37.5%), eravacycline (12.4%), cefiderocol (10.9%), meropenem-vaborbactam (4.7%), omadacycline (1.1%), and imipenem-cilastatin-relebactam (0.9%). In 46% of new antibiotic-prescribing admissions (4647/9768), the new antibiotic was started within the first 3 days of hospital admission. In most cases, new antibiotics were used as definitive therapy (70%, n = 6799).

**Table 1.** New Antibiotics, N = 9768

	N (%)	Number of Courses > 3 Days, N (%)^a^	Days of Therapy (Median, IQR)	Unique Hospitals Prescribing, N = 537, N (%)^b^
Cefiderocol	1060 (10.9)	836 (78.9)	7 (7.5)	202 (37.6)
Ceftazidime-avibactam	3660 (37.5)	2505 (68.4)	6 (6)	454 (84.5)
Ceftolozane-tazobactam	4157 (42.6)	2608 (62.7)	5 (6)	394 (73.4)
Imipenem-cilastatin-relebactam	88 (0.9)	68 (77.2)	7 (9)	47 (8.8)
Meropenem-vaborbactam	456 (4.7)	332 (72.8)	6.5 (8)	128 (23.8)
Eravacycline	1213 (12.4)	852 (70.2)	6 (7)	154 (28.7)
Omadacycline	104 (1.1)	67 (64.4)	5 (5.5)	14 (2.6)

^a^Row percentages.

^b^Column percentages.

Table 2 compares the characteristics of patients treated with new antibiotics to patients who were treated with piperacillin-tazobactam. On average, patients treated with new antibiotics had more comorbidities (median 14 v. 6 Elixhauser comorbidities associated with risk of mortality) and longer length of stay in the hospital (median 13 v. 6 days) than patients treated with piperacillin-tazobactam.

**Table 2.** Demographics of Patients Receiving Newer Antibiotics Compared to Those Receiving Piperacillin-tazobactam

	Newer Antibiotics (N = 9768)	Piperacillin-tazobactam (N = 731 719)	*P* Value^a^
Age, median (IQR)	64 (23)	65 (24)	< .001
Female, n (%)	4230 (43.3)	332 935 (45.5)	< .001 (OR 0.91)
Race, n (%)			
White	6515 (66.7)	535 039 (73.1)	< .001
Black	1934 (19.8)	108 620 (14.8)	
Asian	248 (2.5)	18 480 (2.5)	
Other	701 (7.2)	48 665 (6.7)	
Unknown	370 (3.8)	20 915 (2.9)	
Elixhauser risk of in-hospital mortality, median (IQR)	14 (26)	6 (22)	< .001
Elixhauser Risk of 30-day readmission, median (IQR)	16 (14)	10 (14)	< .001
Solid organ transplant, N (%)	346 (3.5)	8793 (1.2)	< .001 (OR 3.02)
Sepsis^b^	7444 (76.2)	386 744 (52.8)	< .001 (OR 2.86)
All UTI	3781 (38.7)	131 657 (18.0)	< .001 (OR 2.88)
Complicated UTI	2271 (23.2)	57 569 (7.9)	< .001 (OR 3.55)
Pneumonia	4476 (45.8)	207 884 (28.4)	< .001 (OR 2.13)
Abdominal infection	431 (4.4)	24 525 (3.4)	< .001 (OR 1.33)
Length of stay (median, IQR)	13 (19)	6 (7)	< .001

Abbreviations: IQR, interquartile range; OR, odds ratio; UTI, urinary tract infection.

^a^ *t*-test was used to compare age between patient groups. Chi-squared testing was used for gender, race, solid organ transplant, and infection categories. Wilcoxon rank-sum testing was used for Elixhauser indices and length of stay.

^b^Infectious diagnoses were not mutually exclusive. Diagnosis of sepsis included explicit diagnosis of sepsis or implicit diagnosis based on presence of infection plus organ dysfunction.

**Figure 2.**
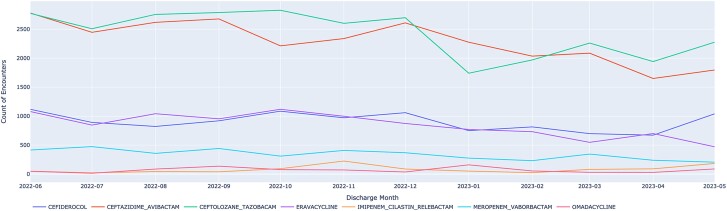
Trends in new antibiotic prescriptions over time.

Table 3 shows the clinical indications associated with use of new antibiotics. Sepsis was the most common clinical indication (76% of new antibiotic prescribing admissions), followed by pneumonia (46%) and urinary tract infection (39%). New antibiotics were received on 1283 number of admissions with none of the previous infectious clinical indications. Similarly, the most common indications associated with treatment with piperacillin-tazobactam were sepsis (53%), pneumonia (28%), and urinary tract infection (18%). Figure 2 shows trends in the prescribing of new antibiotics over time.

**Table 3.** Categories of Diagnoses^a^

	New Antibiotics (N = 9768)	Ceftolozane-tazobactam (N = 4157)	Ceftazidime-avibactam (N = 3660)	Piperacillin-tazobactam (N = 731 719)
Sepsis	7444 (76.2)	3012 (72.4%)	2936 (80.2%)	386 744 (52.8)
All urinary tract infections	3781 (38.7)	1523 (36.6%)	180 (4.9%)	131 657 (18.0)
Pneumonia	4476 (45.8)	1997 (48.0%)	1720 (47.0%)	207 884 (28.4)
Abdominal infection	431 (4.4)	127 (3.1%)	180 (4.9%)	24 525 (3.4)

^a^Diagnoses were not limited to primary or admitting diagnosis. Thus, patients could have received a code for more than 1 diagnosis.

## DISCUSSION

Ceftazidime-avibactam and ceftolozane-tazobactam remain the most frequently prescribed new antibiotics, with uptake of subsequently approved agents trailing. Most of the prescribing of these antibiotics are for sepsis. Patients prescribed these new antibiotics have more comorbid conditions than patients receiving piperacillin-tazobactam and longer hospital length of stay.

Previous work on the use of new antibiotics is limited. Strich et al. examined use of new Gram-negative antibiotics in the PINC-AI database for the years 2016–2021, though their analysis was limited to hospitals that reported antibiotic susceptibility data. They concluded that clinicians still frequently treat resistant Gram-negative infections with older, traditional antibiotics despite suboptimal safety–efficacy profiles. Clancy et al. described use of new antibiotics with activity against CRE in the IQVIA database for the years 2011–2019. They found that new antibiotics had surpassed intravenous polymyxins as treatment for CRE, but use was lower than expected considering that new antibiotics were frequently positioned as first-line therapy by pharmacists at US hospitals. Neither of these manuscripts analyzed indications for prescription of these new antibiotics. Compared with these prior studies, we observed more prescribing of new antibiotics and less prescribing of polymyxins such as colistin. Given that new antibiotics offer improved efficacy and fewer side effects than polymyxins, the changes we observed likely represent progress [18].

Innovations take a long time to disseminate in health care [19, 20]. Evidence supporting improved clinical outcomes with ceftazidime-avibactam compared to polymyxins has been available for nearly a decade [21, 22]. Multiple factors have likely contributed to the delayed adoption of new antibiotics in real-world practice, including access to validated methods for susceptibility testing and geographic variation in the prevalence of resistance [23–25]. Additionally, newer antibiotics may not offer the complete spectrum of coverage as older antibiotics, such as the anaerobic coverage offered by piperacillin-tazobactam. Though we did not limit our study to hospitals contributing microbiology data and are therefore unable to assess the appropriateness of antibiotic therapy, our finding that most new antibiotics were used more frequently than polymyxins suggests that practice is finally catching up to the evidence. Nonetheless, cefiderocol, meropenem-vaborbactam, and imipenem-cilastatin-relebactam are still rarely used.

On the other hand, expanding usage of new antibiotics can potentially have intrinsic risks, including (1) worsening spread of antimicrobial resistance to these last-line antibiotics, (2) high drug costs, and (3) new antibiotics can sometimes be less effective in actual clinical practice than in clinical trials or in vitro studies (eg, tigecycline, trovafloxacin).

Antimicrobial resistance is a national concern, and the lack of national antibiotic use data represents a major gap in public health surveillance. We conducted this study with a commercially available database, PINC-AI. However, national databases that collect and share antibiotic use data, such as the Centers for Disease Control and Prevention National Healthcare Safety Network antibiotic use module [26, 27], are needed. Our hope is that, in the future, publicly available databases of antibiotic use will make studies like this one bigger, cheaper, and more interesting. To enable these future studies, databases will need to adopt standard methodologies for recording for antibiotic use data, such as the WHO GLASS [28].

### Limitations

We estimated antibiotic use based on daily hospital charges and administration data. Not all medications that are charged are necessarily administered to patients. We did not review individual patient records or analyze antibiotic susceptibility data and thus do not know if new antibiotics were used appropriately. However, the advantage of our approach is that most patients are treated with antibiotic therapy in the absence of culture data, and our results are thus more broadly generalizable. A limitation of our manuscript is that we did not assess a temporal change in new Gram-negative antibiotics utilizing the same database over different calendar years.

## CONCLUSION

As antibiotic resistance continues to evolve, so does clinical practice. Though new Gram-negative antibiotics are not used nearly as frequently as workhorses like piperacillin-tazobactam, their use has largely surpassed less effective and more toxic antibiotics like colistin. However, the newest antibiotics, such as cefiderocol, meropenem-vaborbactam, and imipenem-cilastatin-relebactam, remain rare in clinical practice.

## Supplementary Material

ofaf079_Supplementary_Data

## Supplementary Data

Supplementary materials are available at *Open Forum Infectious Diseases* online. Consisting of data provided by the authors to benefit the reader, the posted materials are not copyedited and are the sole responsibility of the authors, so questions or comments should be addressed to the corresponding author.

## Notes

***Financial support.*** J.D.B. received funding support from the Agency for Healthcare Research and Quality (K08HS028854).
