# Supplementary material for: New Kids on the Block: Estimating Use of Next-generation Gram-negative Antibiotics Across Greater Than 700 Hospitals in the United States
Source: Open Forum Infect Dis. 2025 Feb 12;12(2):ofaf079. doi: 10.1093/ofid/ofaf079 (PMC11878548; doi:10.1093/ofid/ofaf079)
Supplement: ofaf079_Supplementary_Data [file ofaf079_supplementary_data.docx]

**APPENDIX**

**SEPSIS (includes explicit or implicit sepsis)**

**Explicit sepsis^1^**

A021, A227, A267, A327, A400, A401, A403, A408, A409, A4101, A4102, A411, A412, A413, A414, A4150, A4151, A4152, A4153, A4159, A4181, A4189, A419, A427, A5486, B377, R6520, R6521

**Implicit sepsis (infection plus organ dysfunction)**

**ICD-10 infection codes (892 codes)**^2^

A000, A001, A009, A0100, A011, A012, A013, A014, A020, A021, A0220, A0221, A0222, A0223, A0224, A0229, A028, A029, A030, A031, A032, A033, A038, A039, A050, A051, A052, A058, A053, A055, A054, A059, A044, A040, A041, A042, A043, A044, A048, A045, A046, A0471, A0472, A049, A080, A082, A0811, A0819, A0831, A0832, A0839, A088, A048, A048, A048, A048, A049, A09, A170, A171, A1781, A1782, A1789, A179, A192, A198, A199, A200, A201, A207, A202, A208, A209, A210, A213, A212, A211, A217, A218, A219, A220, A221, A222, A227, A228, A229, A230, A231, A232, A233, A238, A239, A240, A243, A249, A250, A251, A259, A3211, A3212, A327, A3281, A3289, A329, A267, A268, A269, A280, A288, A289, A360, A361, A3689, A362, A3686, A3681, A3689, A3685, A363, A3682, A3683, A3684, A3689, A369, A3700, A3710, A3780, A3790, J020, J0300, A389, A46, A390, A3981, A394, A391, A3950, A3953, A3951, A3952, A3982, A3983, A3989, A399, A35, A409, A412, A4101, A4102, A411, A403, A414, A4150, A413, A4151, A4152, A4153, A4159, A4189, A419, L081, A420, A421, A422, B479, A4281, A4282, A4289, A438, A429, A439, B471, A480, A488, K9081, A4851, A4852, M60009, A483, B955, B950, B951, B954, B952, B958, B9561, B9562, B957, B953, B961, B9621, B9622, B9623, B9620, B9629, B963, B964, B965, A493, B960, B966, B967, B9689, B9681, A5400, A5429, A5401, A5422, A5423, A5423, A5403, A5424, A5429, A5421, A5431, A5432, A5439, A5433, A5442, A5449, A5441, A5440, A545, A546, A5489, A5481, A5483, A5485, A5486, A270, A2781, A2789, A279, A690, A691, B370, B3783, B373, B3742, B3749, B372, B371, B377, B376, B3784, B375, B3781, B3782, B3789, B379, B371, B377, B376, B3784, B375, B3781, B3782, B3789, B380, B383, B384, B3889, B381, B382, B389, B39, G02, H32, I32, I39, J17, B40, B41, B480, B481, B42, B43, B44, B470, B450, B457, B459, B482, B469, B488, B49, B488, G000, G001, G002, G003, G01, G008, G009, G042, B451, G02, G02, G060, G061, G062, G08, H44009, H44019, H44029, H44139, H44119, H33129, H44129, H16249, H4419, H4430, H4420, H4421, H4422, H4423, H442A1, H442A2, H442A3, H442A9, H442B1, H442B2, H442B3, H442B9, H442C1, H442C2, H442C3, H442C9, H442D1, H442D2, H442D3, H442D9, H442E1, H442E2, H442E3, H442E9, H44329, H44319, H44399, H4440, H44449, H44429, H44439, H44419, H4450, H44529, H44519, H44819, H44539, H44609, H44619, H44629, H44639, H44659, H44649, H44699, H44709, H44719, H44729, H44739, H44759, H44749, H44799, H44829, H4489, H449, H0500, H05019, H05039, H05029, H05049, H0510, H05119, H05129, H0589, H0520, H05249, H05239, H05229, H05259, H05269, H05219, H0530, H0589, H05359, H05329, H0589, H05319, H05349, H05339, H05409, H05419, H05429, H0553, H05819, H05829, H0589, H059, H6020, I308, I330, I39, I339, J0100, J0110, J0120, J0130, J0140, J0190, J029, J0390, J040, J050, J0410, J0411, J042, J050, J0510, J0511, J0430, J0431, J060, J069, J36, J13, J181, J150, J151, J14, J154, J153, J1520, J15211, J15212, J1529, J158, J155, J156, A481, J158, J159, J180, J189, J441, J479, J471, J860, J869, J850, J851, J852, J853, K047, K046, M272, K113, K122, K352, K353, K3580, K3589, K37, K36, K5712, K5713, K5732, K5733, K610, K611, K613, K67, K658, K650, K651, K652, K650, K6812, K6819, K689, K653, K654, K658, K659, K630, K9402, K631, K750, K751, K7290, K7291, K766, K767, K7210, K7290, K751, K810, K819, K811, K812, K820, K821, K822, K823, K824, K828, K829, N110, N118, N10, N151, N2884, N2885, N2886, N12, N16, N159, N390, N360, N361, N362, N365, N368, N139, R319, R310, R311, R3121, R3129, N3641, N3642, N368, N398, N369, N399, N410, N411, N412, N413, N51, N414, N418, N419, N454, N451, N452, N453, N51, N7001, N7002, N7003, N7011, N7012, N7013, N7091, N7092, N7093, N730, N731, N732, N733, N736, N734, N738, N739, N710, N711, N719, N751, N764, O030, O0337, O035, O0387, O036, O031, O0384, O0382, O0383, O0333, O0381, O037, O032, O0385, O0386, O0388, O0389, O0339, O0385, O0380, O0330, O039, O034, O045, O0487, O046, O0484, O0482, O0483, O0481, O047, O0485, O0486, O0488, O0489, O0480, Z332, O045, O046, O0484, O0482, O0483, O0481, O047, O0489, O0480, Z332, O045, O046, O0484, O0482, O0483, O0481, O047, O0489, O0480, Z332, O070, O0737, O071, O0734, O0732, O0733, O0731, O072, O0735, O0736, O0738, O0739, O0730, O074, A34, O080, O0882, O081, O086, O084, O085, O083, O082, O087, O0881, O0883, O0889, O089, O2300, O2310, O2320, O2330, O2340, O23519, O23529, O23599, O2390, O2391, O2392, O2393, O8611, O8613, O8619, O8620, O8621, O8622, O8629, O411090, O411290, O411490, O411010, O411020, O411030, O411210, O411220, O411230, O411410, O411420, O411430, O8689, O8612, O8681, O91119, O91111, O91112, O91113, O9112, L03019, L03029, L03039, L03049, K122, L03211, L03212, L03213, L03221, L03222, L03319, L03329, L03119, L03129, L03317, L03811, L03818, L03891, L03898, L0390, L0391, L049, L0501, L0502, L05091, L0592, L080, L88, L0889, L980, E832, L0889, L089, M0000, M0010, M0020, M0080, M009, M00019, M00119, M00219, M00819, M00029, M00129, M00229, M00829, M00039, M00139, M00239, M00839, M00049, M00149, M00249, M00849, M00059, M00159, M00259, M00859, M00069, M00169, M00269, M00869, M00079, M00179, M00279, M00879, M0008, M0018, M0028, M0088, M009, M0009, M0019, M0029, M0089, M0230, M02319, M02329, M02339, M02349, M02359, M02369, M02379, M0238, M0239, M352, M0210, M02119, M02129, M02139, M02149, M02159, M02169, M02179, M0218, M0219, M01X0, M01X19, M01X29, M01X39, M01X49, M01X59, M01X69, M01X79, M01X8, M01X9, M726, M8610, M8620, M86119, M86219, M86129, M86229, M86139, M86239, M86149, M86249, M86159, M86259, M86169, M86269, M86179, M86279, M8618, M8628, M8619, M8629, M8660, M86619, M86629, M86639, M86642, M86659, M86669, M86679, M8668, M8669, M869, M4620, M8960, M89619, M89629, M89639, M89649, M89659, M89669, M89679, M8968, M8969, M9080, M90819, M90829, M90839, M90849, M90859, M90869, M90879, M9088, M9089, M4630, R7881, T798XXA, T8579XA, T826XXA, T827XXA, T827XXA, T85730A, T85731A, T85732A, T85733A, T85734A, T85735A, T85738A, T83510A, T83511A, T83512A, T83518A, T83590A, T83591A, T83592A, T83593A, T83598A, T8361XA, T8362XA, T8369XA, T8450XA, T8460XA, T847XXA, T8571XA, T8579XA, T814XXA, K6811, T814XXA, T80219A, T80211A, T80212A, T8022XA, T8029XA, T880XXA

**ICD-10 organ dysfunction codes**^3^

R57.x, I95.1, I95.89-9, R03.1, R65.21, I46.9, J96.0x, J96.9x, J80, R06.00, R06.03, R06.09, R06.3, R06.83, R06.89, R09.2, F05, G93.1, G93.40, R40.1-2, D65, D68.8-9, D69.59-6, K72.00-01, K72.91, K76.2-3, N17.x

**UTI/COMPLICATED UTI**^4^

1. At least One Diagnosis from Group A OR At least One Diagnosis from Group B AND At least One Event (Either Diagnosis or Procedure) from Group C
2. Presence of any code from Group A as a primary or secondary diagnosis identifies a UTI infection.
3. To identify a cUTI infection, the diagnosis must appear on an inpatient claim.
4. Presence of any code from Group B as a primary or secondary diagnosis identifies a UTI.
   1. To identify a cUTI, a code from Group B must appear along with a diagnosis or procedure code from Group C.
5. Presence of any code from Group C as a primary or secondary diagnosis or procedure, in addition to a code from Group B, identifies a cUTI.

**Group A ICD-10-CM codes**

N10, N11.0, N11.8, N12, N15.1, N15.9, N16, N28.84, N28.85, N28.86, N35.111, N35.112, N35.113, N35.114, N35.119, N35.12, T83.510A, T83.511A, T83.512A, T83.518A

**Group B ICD-10-CM codes**

N13.9, N30.00, N30.01, N30.10, N30.11, N30.20, N30.21, N30.30, N30.31, N30.40, N30.41, N30.80, N30.81, N30.90, N30.91, N34.0, N34.1, N34.2, N34.3, N35.014, N35.028, N35.1, N35.8, N35.9, N36.0, N36.1, N36.2, N36.5, N36.8, N99.110, N37, N39

**Group C**

**CPT-4 Codes:**

57102, 57103

**ICD-10 PCS Codes:**

0T9B70Z, 0T9B80Z, 0T2BX0Z, 3C1ZX8Z

**ICD-10-CM Codes:**

B08.70, B08.71, Z46.6 B08.72, N13.30, N13.39, N13.9, N20.0, N20.1, N20.2, N20.9, N31.2, N31.9, N32.0, N32.89, N32.9, N36.44, N40.0, N40.1, N40.2, N40.3, N42.83, N99.510, N99.511, N99.512, N99.518, Q62.10, Q62.11, Q62.12, Q62.31, Q62.39, R33.8, R33.9, R39.14, Z43.6

**PNEUMONIA**^5^

**ICD-10 Codes (mapped from ICD-9 codes**^6^**)**

B371, B380, B392, B395, J17, B399, J17, B583, B59, J120, J121, J122, J1289, J129, J13, J181, J150, J151, J14, J159, B250, A3791, A221, B440, J17, J17, J180, J189, J690, J860, J869, J90, J942, J850, J851, J852, J95851

**ABDOMINAL INFECTION**^7^

Criteria for diagnosis of a complicated intra-abdominal infection ≥1 ICE-10 diagnosis code from Group A and ≥1 ICD-10 procedure code from Group B

**Group A ICD-10 Codes**

K57.12, K57.13, K57.32, K57.33, K63.2, K63.3, K63.1 K25.1, K56.60, K25.2, K56.60, K25.5, K56.60, K25.6, K26.1, K26.2, K26.5, K26.6, K27.1, K2.72, K27.5, K27.6, K27.6, K28.1, K28.2, K28.5, K28.6, K35.2, K35.3, K37, K36, K67, K65.8, K65.0, K65.1, K65.2, K65.0, K68.12, K68.19, K68.9, K6.53, K65.4, K65.8, K65.9, K63.0, K75.0, K75.1, K72.90, K72.91, K76.6, K76.7, K72.10, K72.90, K82.2 plus (K80.00, K80.01, K80.42, K80.43, K80.62, K80.63, K80.66, or K80.67), K81.0, K83.0

**Group B ICD-10 Procedure Codes**

0DB40ZZ, 0DB43ZZ, 0DB44ZZ, 0DB47ZZ, 0DT40ZZ, 0DT44ZZ, 0DT47ZZ, 0DT48ZZ, 0DB60ZZ, 0DB63ZZ, 0DB67ZZ, 0DT70ZZ, 0DT74ZZ, 0DT77ZZ, 0DT78ZZ, 0D160ZA, 0D164ZA, 0D168ZA, 0DB60ZZ, 0DB63ZZ, 0DB64ZZ, 0DB67ZZ, 0DB68ZZ, 0D160ZA, 0D164ZA, 0D168ZA, 0DB60ZZ, 0DB63ZZ, 0DB64ZZ, 0DB67ZZ, 0DB68ZZ, 0DB64Z3, 0DB60ZZ, 0DB63ZZ, 0DB67ZZ, 0D13079, 0D1307A, 0D1307B, 0DT60ZZ, 0DT64ZZ, 0DT67ZZ, 0DT68ZZ, 0DT60ZZ, 0DT64ZZ, 0DT67ZZ, 0DT68ZZ, 0DQ60ZZ, 0DQ63ZZ, 0DQ64ZZ, 0DQ67ZZ, 0DQ68ZZ, 0DQ90ZZ, 0DQ93ZZ, 0DQ94ZZ, 0DQ97ZZ, 0DQ98ZZ, 0DQ60ZZ, 0DQ63ZZ, 0DQ64ZZ, 0DQ67ZZ, 0DQ68ZZ, 0DQ90ZZ, 0DQ93ZZ, 0DQ94ZZ, 0DQ97ZZ, 0DQ98ZZ, 0DQ60ZZ, 0DQ63ZZ, 0DQ64ZZ, 0DQ67ZZ, 0DQ68ZZ, 0DB80ZZ, 0DB83ZZ, 0DB84ZZ, 0DB87ZZ, 0DB88ZZ, 0DT90ZZ, 0DT94ZZ, 0DT97ZZ, 0DT98ZZ, 0DTA0ZZ, 0DTA4ZZ, 0DTA7ZZ, 0DTA8ZZ, 0DTB0ZZ, 0DTB4ZZ, 0DTB7ZZ, 0DTB8ZZ, 0DT80ZZ, 0DT84ZZ, 0DT87ZZ, 0DT88ZZ, 0D1H0Z4, 0D1H4Z4, 0D1H8Z4, 0D1K0Z4, 0D1K4Z4, 0D1K8Z4, 0D1L0Z4, 0D1L4Z4, 0D1L8Z4, 0D1N0Z4, 0D1N4Z4, 0D1N8Z4, 0D1B0Z4, 0D1B4Z4, 0D1B8Z4, 0D1B0Z4, 0D1B4Z4, 0D1B8Z4, 0D1B0Z4, 0D1B4Z4, 0D1B8Z4, 0D1B0Z4, 0D1B4Z4, 0D1B8Z4, 0DQ90ZZ, 0DQ93ZZ, 0DQ94ZZ, 0DQ97ZZ, 0DQ98ZZ, 0DQ90ZZ, 0DQ93ZZ, 0DQ94ZZ, 0DQ97ZZ, 0DQ98ZZ, 0DQ80ZZ, 0DQ83ZZ, 0DQ84ZZ, 0DQ87ZZ, 0DQ88ZZ, 0DQA0ZZ, 0DQA3ZZ, 0DQA4ZZ, 0DQA7ZZ, 0DQA8ZZ, 0DQB0ZZ, 0DQB3ZZ, 0DQB4ZZ, 0DQB7ZZ, 0DQB8ZZ, 0DQ80ZZ, 0DQ80ZZ, 0DQ83ZZ, 0DQ83ZZ, 0DQ84ZZ, 0DQ84ZZ, 0DQ87ZZ, 0DQ87ZZ, 0DQ88ZZ, 0DQ88ZZ, 0DQA0ZZ, 0DQA3ZZ, 0DQA4ZZ, 0DQA7ZZ, 0DQA8ZZ, 0DQB0ZZ, 0DQB0ZZ, 0DQB3ZZ, 0DQB3ZZ, 0DQB4ZZ, 0DQB4ZZ, 0DQB7ZZ, 0DQB7ZZ, 0DQB8ZZ, 0DQB8ZZ, 0DQE0ZZ, 0DQE3ZZ, 0DQE4ZZ, 0DQE7ZZ, 0DQE8ZZ, 0DQN0ZZ, 0DQN3ZZ, 0DQN4ZZ, 0DQN7ZZ, 0DQN8ZZ, 0DQP0ZZ, 0DQP3ZZ, 0DQP4ZZ, 0DQP7ZZ, 0DQP8ZZ, 0HQ6XZZ, 0HQ7XZZ, 0DQE0ZZ, 0DQE3ZZ, 0DQE4ZZ, 0DQE7ZZ, 0DQE8ZZ, 0DQH0ZZ, 0DQH3ZZ, 0DQH4ZZ, 0DQH7ZZ, 0DQH8ZZ, 0DQK0ZZ, 0DQK3ZZ, 0DQK4ZZ, 0DQK7ZZ, 0DQK8ZZ, 0DQN0ZZ, 0DQN3ZZ, 0DQN4ZZ, 0DQN7ZZ, 0DQN8ZZ, 0DQE0ZZ, 0DQE3ZZ, 0DQE4ZZ, 0DQE7ZZ, 0DQE8ZZ, 0DQH0ZZ, 0DQH3ZZ, 0DQH4ZZ, 0DQH7ZZ, 0DQH8ZZ, 0DQN0ZZ, 0DQN0ZZ, 0DQN3ZZ, 0DQN3ZZ, 0DQN4ZZ, 0DQN4ZZ, 0DQN7ZZ, 0DQN7ZZ, 0DQN8ZZ, 0DQN8ZZ, 0HQ9XZZ, 0DQ90ZZ, 0DQ93ZZ, 0DQ94ZZ, 0DQ97ZZ, 0DQ98ZZ, 0DQE0ZZ, 0DQE3ZZ, 0DQE4ZZ, 0DQE7ZZ, 0DQE8ZZ, 0DS90ZZ, 0DS94ZZ, 0DS97ZZ, 0DS98ZZ, 0DSA0ZZ, 0DSA4ZZ, 0DSA7ZZ, 0DSA8ZZ, 0DSB0ZZ, 0DSB4ZZ, 0DSB7ZZ, 0DSB8ZZ, 0DSH0ZZ, 0DSH4ZZ, 0DSH7ZZ, 0DSH8ZZ, 0DSK0ZZ, 0DSK4ZZ, 0DSK7ZZ, 0DSK8ZZ, 0DSL0ZZ, 0DSL4ZZ, 0DSL7ZZ, 0DSL8ZZ, 0DSM0ZZ, 0DSM4ZZ, 0DSM7ZZ, 0DSM8ZZ, 0DSN0ZZ, 0DSN4ZZ, 0DSN7ZZ, 0DSN8ZZ, 0DS90ZZ, 0DS94ZZ, 0DS97ZZ, 0DS98ZZ, 0DSA0ZZ, 0DSA4ZZ, 0DSA7ZZ, 0DSA8ZZ, 0DSB0ZZ, 0DSB4ZZ, 0DSB7ZZ, 0DSB8ZZ, 0DSH0ZZ, 0DSH4ZZ, 0DSH7ZZ, 0DSH8ZZ, 0DSK0ZZ, 0DSK4ZZ, 0DSK7ZZ, 0DSK8ZZ, 0DSL0ZZ, 0DSL4ZZ, 0DSL7ZZ, 0DSL8ZZ, 0DSM0ZZ, 0DSM4ZZ, 0DSM7ZZ, 0DSM8ZZ, 0DSN0ZZ, 0DSN4ZZ, 0DSN7ZZ, 0DSN8ZZ, 0D7N0ZZ, 0D7N3ZZ, 0D7N4ZZ, 0D780ZZ, 0D783ZZ, 0D784ZZ, 0D7E0ZZ, 0D7E3ZZ, 0D7E4ZZ, 0DQ80ZZ, 0DQ83ZZ, 0DQ84ZZ, 0DQ87ZZ, 0DQ88ZZ, 0DQA0ZZ, 0DQA3ZZ, 0DQA4ZZ, 0DQA7ZZ, 0DQA8ZZ, 0DQB0ZZ, 0DQB3ZZ, 0DQB4ZZ, 0DQB7ZZ, 0DQB8ZZ, 0DQE0ZZ, 0DQE3ZZ, 0DQE4ZZ, 0DQE7ZZ, 0DQE8ZZ, 0DQ83ZZ, 0DQ84ZZ, 0DQ87ZZ, 0DQ88ZZ, 0DQE0ZZ, 0DQE3ZZ, 0DQE4ZZ, 0DQE7ZZ, 0DQE8ZZ, 0DTJ4ZZ, 0DTJ0ZZ, 0DTJ7ZZ, 0DTJ8ZZ, 0DTJ4ZZ, 0DTJ0ZZ, 0DTJ7ZZ, 0DTJ8ZZ, 0D9J00Z, 0D9J0ZZ, 0D9J30Z, 0D9J3ZZ, 0D9J40Z, 0D9J4ZZ, 0D9J70Z, 0D9J7ZZ, 0D9J80Z, 0D9J8ZZ, 0D9J00Z, 0D9J0ZZ, 0D9J30Z, 0D9J3ZZ, 0D9J40Z, 0D9J4ZZ, 0D9J70Z, 0D9J7ZZ, 0D9J80Z, 0D9J8ZZ, 0DQJ0ZZ, 0DQJ0ZZ, 0DQJ3ZZ, 0DQJ3ZZ, 0DQJ4ZZ, 0DQJ4ZZ, 0DQJ7ZZ, 0DQJ7ZZ, 0DQJ8ZZ, 0DQJ8ZZ, 0HQ6XZZ, 0HQ7XZZ, 0DQJ0ZZ, 0DQJ3ZZ, 0DQJ4ZZ, 0DQJ7ZZ, 0DQJ8ZZ 0F9000Z, 0F900ZZ, 0FC00ZZ, 0FC03ZZ, 0FC04ZZ, 0F900ZX, 0FB00ZX, 0FB03ZX, 0FB04ZX, 0FJ03ZZ, 0F900ZZ, 0F903ZZ, 0F904ZZ, 0FB00ZZ, 0FB03ZZ, 0FB04ZZ, 0F500ZZ, 0F503ZZ, 0F504ZZ, 0F500ZZ, 0F500ZZ, 0F503ZZ, 0F504ZZ, 0FT10ZZ, 0FT14ZZ, 0FT20ZZ, 0FT24ZZ, 0FT00ZZ, 0FT04ZZ, 0FY00Z0, 0FY00Z1, 0FY00Z2, 0FY00Z0, 0FY00Z1, 0FY00Z2, 0FQ00ZZ, 0FQ03ZZ, 0FQ04ZZ, 0FQ00ZZ, 0FQ03ZZ, 0FQ04ZZ, 0FS00ZZ, 0FS04ZZ, 0F9430Z, 0F940ZZ, 0F9400Z, 0FC40ZZ, 0FC43ZZ, 0FC44ZZ, 0FF40ZZ, 0FF43ZZ, 0FF44ZZ, 0FF47ZZ, 0F940ZX, 0F950ZX, 0F960ZX, 0F980ZX, 0F990ZX, 0F9C0ZX, 0F9D0ZX, 0FB40ZX, 0FB50ZX, 0FB60ZX, 0FB80ZX, 0FB90ZX, 0FBC0ZX, 0FB40ZZ, 0FB43ZZ, 0FT40ZZ, 0FT44ZZ, 0FB44ZZ, 0F140D5, 0F140D6, 0F140D7, 0F140Z5, 0F140Z6, 0F140Z7, 0F144D5, 0F144D6, 0F144D7, 0F144Z5, 0F144Z6, 0F144Z7, 0F140D3, 0F140DB, 0F140Z3, 0F140ZB, 0F144D3, 0F144DB, 0F144Z3, 0F144ZB, 0F140D4, 0F140Z4, 0F144D4, 0F144Z4, 0F140D8, 0F140D9, 0F140Z8, 0F140Z9, 0F144D8, 0F144D9, 0F144Z8, 0F144Z9, 0F190D3, 0F190Z3, 0F194D3, 0F194Z3, 0F150D3, 0F150DB, 0F150Z3, 0F150ZB, 0F154D3, 0F154DB, 0F154Z3, 0F154ZB, 0F160D3, 0F160DB, 0F160Z3, 0F160ZB, 0F164D3, 0F164DB, 0F164Z3, 0F164ZB, 0F180D3, 0F180DB, 0F180Z3, 0F180ZB, 0F184D3, 0F184DB, 0F184Z3, 0F184ZB, 0F190DB, 0F190ZB, 0F194DB, 0F194ZB, 0F150D5, 0F150D6, 0F150D7, 0F150D8, 0F150D9, 0F150Z5, 0F150Z6, 0F150Z7, 0F150Z8, 0F150Z9, 0F154D5, 0F154D6, 0F154D7, 0F154D8, 0F154D9, 0F154Z5, 0F154Z6, 0F154Z7, 0F154Z8, 0F154Z9, 0F160D5, 0F160D6, 0F160D7, 0F160D8, 0F160D9, 0F160Z5, 0F160Z6, 0F160Z7, 0F160Z8, 0F160Z9, 0F164D5, 0F164D6, 0F164D7, 0F164D8, 0F164D9, 0F164Z5, 0F164Z6, 0F164Z7, 0F164Z8, 0F164Z9, 0F180D4, 0F180D5, 0F180D6, 0F180D7, 0F180D8, 0F180D9, 0F180Z4, 0F180Z5, 0F180Z6, 0F180Z7, 0F180Z8, 0F180Z9, 0F184D4, 0F184D5, 0F184D6, 0F184D7, 0F184D8, 0F184D9, 0F184Z4, 0F184Z5, 0F184Z6, 0F184Z7, 0F184Z8, 0F184Z9, 0F190D4, 0F190D5, 0F190D6, 0F190D7, 0F190D8, 0F190D9, 0F190Z4, 0F190Z5, 0F190Z6, 0F190Z7, 0F190Z8, 0F190Z9, 0F194D4, 0F194D5, 0F194D6, 0F194D7, 0F194D8, 0F194D9, 0F194Z4, 0F194Z5, 0F194Z6, 0F194Z7, 0F194Z8, 0F194Z9, 0FC90ZZ, 0FC90ZZ, 0F9970Z, 0FC50ZZ, 0FC60ZZ, 0FC80ZZ, 0FF50ZZ, 0FF53ZZ, 0FF54ZZ, 0FF57ZZ, 0FF60ZZ, 0FF63ZZ, 0FF64ZZ, 0FF67ZZ, 0FF80ZZ, 0FF83ZZ, 0FF84ZZ, 0FF87ZZ, 0FF90ZZ, 0FF93ZZ, 0FF94ZZ, 0FF97ZZ, 0FFC0ZZ, 0FFC3ZZ, 0FFC4ZZ, 0FFC7ZZ, 0F9900Z, 0F990ZZ, 0F9930Z, 0F9940Z, 0FJB0ZZ, 0FJB3ZZ, 0FJB4ZZ, 0FJB7ZZ, 0FJB8ZZ, 0F9500Z, 0F950ZZ, 0F9530Z, 0F953ZZ, 0F9540Z, 0F954ZZ, 0F9570Z, 0F957ZZ, 0F9580Z, 0F958ZZ, 0F9600Z, 0F960ZZ, 0F9630Z, 0F963ZZ, 0F9640Z, 0F964ZZ, 0F9670Z, 0F967ZZ, 0F9680Z, 0F968ZZ, 0F9800Z, 0F980ZZ, 0F9830Z, 0F983ZZ, 0F9840Z, 0F984ZZ, 0F9870Z, 0F987ZZ, 0F9880Z, 0F988ZZ, 0FHB0DZ, 0FHB3DZ, 0FHB7DZ, 0FJB0ZZ, 0FB80ZZ, 0FB83ZZ, 0FB87ZZ, 0FBC0ZZ, 0FBC3ZZ, 0FBC7ZZ, 0FTC0ZZ, 0FTC4ZZ, 0FTC7ZZ, 0FTC8ZZ, 0FB90ZZ, 0FB93ZZ, 0FB97ZZ, 0FT90ZZ, 0FT94ZZ, 0FT97ZZ, 0FT98ZZ 0F550ZZ, 0F553ZZ, 0F557ZZ, 0F560ZZ, 0F563ZZ, 0F567ZZ, 0F580ZZ, 0F583ZZ, 0F587ZZ, 0FB50ZZ, 0FB53ZZ, 0FB57ZZ, 0FB60ZZ, 0FB63ZZ, 0FB67ZZ, 0FB80ZZ, 0FB83ZZ, 0FB87ZZ, 0FT50ZZ, 0FT54ZZ, 0FT57ZZ, 0FT58ZZ, 0FT60ZZ, 0FT64ZZ, 0FT67ZZ, 0FT68ZZ, 0FT80ZZ, 0FT84ZZ, 0FT87ZZ, 0FT88ZZ 0FQ90ZZ, 0FQ93ZZ, 0FQ94ZZ, 0FQ97ZZ, 0FQ98ZZ, 0FQ90ZZ, 0FQ93ZZ, 0FQ94ZZ, 0FQ97ZZ, 0FQ98ZZ, 0FQ50ZZ, 0FQ53ZZ, 0FQ54ZZ, 0FQ57ZZ, 0FQ58ZZ, 0FQ60ZZ, 0FQ63ZZ, 0FQ64ZZ, 0FQ67ZZ, 0FQ68ZZ, 0FQ80ZZ, 0FQ83ZZ, 0FQ84ZZ, 0FQ87ZZ, 0FQ88ZZ, 0F7C0DZ, 0F7C0ZZ, 0F7C3DZ, 0F7C3ZZ, 0F7C4DZ, 0F7C4ZZ, 0F7C7DZ, 0F7C7ZZ, 0F8G0ZZ, 0F8G3ZZ, 0FCC0ZZ, 0FQC0ZZ, 0FQC3ZZ, 0FQC4ZZ, 0FQC7ZZ, 0FQC8ZZ, 0FQC0ZZ, 0FQC3ZZ, 0FQC4ZZ, 0FQC7ZZ, 0FQC8ZZ, 0FQ40ZZ, 0FQ43ZZ, 0FQ44ZZ, 0FQ40ZZ, 0FQ43ZZ, 0FQ44ZZ, 0WQFXZ2, 0DQ60ZZ, 0DQ60ZZ, 0DQ63ZZ, 0DQ63ZZ, 0DQ64ZZ, 0DQ64ZZ, 0DQ67ZZ, 0DQ67ZZ, 0DQ68ZZ, 0DQ68ZZ, 0DQ80ZZ, 0DQ80ZZ, 0DQ83ZZ, 0DQ83ZZ, 0DQ84ZZ, 0DQ84ZZ, 0DQ87ZZ, 0DQ87ZZ, 0DQ88ZZ, 0DQ88ZZ, 0DQ90ZZ, 0DQ93ZZ, 0DQ94ZZ, 0DQ97ZZ, 0DQ98ZZ, 0DQA0ZZ, 0DQA3ZZ, 0DQA4ZZ, 0DQA7ZZ, 0DQA8ZZ, 0DQE0ZZ, 0DQE3ZZ, 0DQE4ZZ, 0DQE7ZZ, 0DQE8ZZ, 0FQ40ZZ, 0FQ40ZZ, 0FQ40ZZ, 0FQ43ZZ, 0FQ43ZZ, 0FQ43ZZ, 0FQ44ZZ, 0FQ44ZZ, 0FQ44ZZ, 0FQ50ZZ, 0FQ53ZZ, 0FQ54ZZ, 0FQ57ZZ, 0FQ58ZZ, 0FQ60ZZ, 0FQ63ZZ, 0FQ64ZZ, 0FQ67ZZ, 0FQ68ZZ, 0FQ80ZZ, 0FQ83ZZ, 0FQ84ZZ, 0FQ87ZZ, 0FQ88ZZ, 0FQ90ZZ, 0FQ93ZZ, 0FQ94ZZ, 0FQ97ZZ, 0FQ98ZZ, 0FP40DZ, 0FP43DZ, 0FP44DZ, 0FR50JZ, 0FR54JZ, 0FR60JZ, 0FR64JZ, 0FR80JZ, 0FR84JZ, 0FR90JZ, 0FR94JZ, 0FS40ZZ, 0FS44ZZ, 0F9D00Z, 0F9D30Z, 0F9D40Z, 0F9D70Z, 0F9G00Z, 0F9G30Z, 0F9G40Z, 0F9D0ZZ, 0F9D3ZZ, 0F9D4ZZ, 0F9D7ZZ, 0F9D8ZZ, 0F9G0ZZ, 0F9G3ZZ, 0F9G4ZZ, 0FCD0ZZ, 0FCD7ZZ, 0FCG0ZZ, 0FCG3ZZ, 0FCG4ZZ, 0FFD0ZZ, 0FFD3ZZ, 0FFD4ZZ, 0FFD7ZZ, 0FFD8ZZ, 0F9G0ZX, 0FBG0ZX, 0F5D0ZZ, 0F5D3ZZ, 0F5D7ZZ, 0F5G0ZZ, 0F5G3ZZ, 0FBD0ZZ, 0FBD3ZZ, 0FBD7ZZ, 0FBG0ZZ, 0FBG3ZZ, 0FTD0ZZ, 0FTD7ZZ, 0F9G3ZZ, 0F9G4ZZ, 0F1D0D3, 0F1D0DB, 0F1D0Z3, 0F1D0ZB, 0F1D4D3, 0F1D4DB, 0F1D4Z3, 0F1D4ZB, DB90ZZ, 0DB93ZZ, 0DB94ZZ, 0DB97ZZ, 0DB98ZZ, 0FBG0ZZ, 0FBG0ZZ, 0FBG3ZZ, 0FBG3ZZ, 0FBG4ZZ, 0FBG4ZZ, 0FBG0ZZ, 0FBG3ZZ, 0FBG4ZZ, 0FBG0ZZ, 0FBG3ZZ, 0FBG4ZZ, 0FBG0ZZ, 0FBG3ZZ, 0FBG4ZZ, 0DT90ZZ, 0DT94ZZ, 0DT97ZZ, 0DT98ZZ, 0FTG0ZZ, 0FTG4ZZ, 0D1607A, 0D160JA, 0D160KA, 0D160ZA, 0DT90ZZ, 0DT90ZZ, 0F190Z3, 0F1G0ZC, 0FTG0ZZ, 0FTG0ZZ, 0FYG0Z0, 0FYG0Z1, 0FYG0Z2, 0FSG0ZZ, 0FSG4ZZ, 0FYG0Z0, 0FYG0Z1, 0FYG0Z2, 0F7D0DZ, 0F7D3DZ, 0F7D7DZ, 0FHD0DZ, 0FHD3DZ, 0FHD7DZ, 0FUD37Z, 0FUD47Z, 0FQG0ZZ, 0FQG3ZZ, 0FQG4ZZ, 0F1D0D3, 0F1D0DB, 0F1D0Z3, 0F1D0ZB, 0F1D4D3, 0F1D4DB, 0F1D4Z3, 0F1D4ZB, 0F1G0D3, 0F1G0DB, 0F1G0Z3, 0F1G0ZB, 0F1G4D3, 0F1G4DB, 0F1G4Z3, 0F1G4ZB, 0F7D3ZZ, 0FQD0ZZ, 0FQD3ZZ, 0FQD4ZZ, 0FQD7ZZ, 0FQD8ZZ, 0DJ00ZZ, 0DJ60ZZ, 0DJD0ZZ, 0DJU0ZZ, 0DJW0ZZ, 0WJG0ZZ, 0WJJ0ZZ, 0WJP0ZZ, 0WJR0ZZ, 0WJF4ZZ, 0WJG4ZZ, 0WJJ4ZZ, 0WJP4ZZ, 0WJR4ZZ, 0D5S0ZZ, 0D5S3ZZ, 0D5S4ZZ, 0D5T0ZZ, 0D5T3ZZ, 0D5T4ZZ, 0D5V0ZZ, 0D5V3ZZ, 0D5V4ZZ, 0D5W0ZZ, 0D5W3ZZ, 0D5W4ZZ, 0DBS0ZZ, 0DBS3ZZ, 0DBS4ZZ, 0DBT0ZZ, 0DBT3ZZ, 0DBT4ZZ, 0DBV0ZZ, 0DBV3ZZ, 0DBV4ZZ, 0DBW0ZZ, 0DBW3ZZ, 0DBW4ZZ, 0DTS0ZZ, 0DTS4ZZ, 0DTT0ZZ, 0DTT4ZZ, 0DN84ZZ, 0DNE4ZZ, 0DNJ4ZZ, 0DNS4ZZ, 0DNT4ZZ, 0DNV4ZZ, 0DNW4ZZ, 0FN04ZZ, 0FN44ZZ, 0FN54ZZ, 0FN64ZZ, 0FN84ZZ, 0FN94ZZ, 0FNG4ZZ, 0DNE0ZZ, 0DNE3ZZ, 0DNJ0ZZ, 0DNJ3ZZ, 0DNS0ZZ, 0DNS3ZZ, 0DNT0ZZ, 0DNT3ZZ, 0DNV0ZZ, 0DNV3ZZ, 0DNW0ZZ, 0DNW3ZZ, 0FN00ZZ, 0FN03ZZ, 0FN40ZZ, 0FN43ZZ, 0FN50ZZ, 0FN53ZZ, 0FN57ZZ, 0FN58ZZ, 0FN60ZZ, 0FN63ZZ, 0FN67ZZ, 0FN68ZZ, 0FN80ZZ, 0FN83ZZ, 0FN87ZZ, 0FN88ZZ, 0FN90ZZ, 0FN93ZZ, 0FN97ZZ, 0FN98ZZ, 0FNG0ZZ, 0FNG3ZZ, 0DCS0ZZ, 0DCS3ZZ, 0DCS4ZZ, 0DCT0ZZ, 0DCT3ZZ, 0DCT4ZZ, 0DCV0ZZ, 0DCV3ZZ, 0DCV4ZZ, 0DCW0ZZ, 0DCW3ZZ, 0DCW4ZZ, 0WCG0ZZ, 0WCG3ZZ, 0WCG4ZZ, 0W1G0J4, 0W1G3J4, 0W1G4J4, 0W1G0JY, 0W1G4JY, 0W9J00Z, 0W9J0ZZ, 0W9J40Z, 0W9J4ZZ, 0WWG00Z, 0WWG0JZ, 0WWG30Z, 0WWG3JZ, 0WWG40Z, 0WWG4JZ

**References**

1. Buchman TG, Simpson SQ, Sciarretta KL, et al. Sepsis Among Medicare Beneficiaries: 3. The Methods, Models, and Forecasts of Sepsis, 2012-2018. *Crit Care Med*. 2020;48(3):302-318. doi:10.1097/CCM.0000000000004225

2. Chan HK, Khose S, Chavez S, Patel B, Wang HE. Updated estimates of sepsis hospitalizations at United States academic medical centers. *J Am Coll Emerg Physicians Open*. 2022;3(4):e12782. doi:10.1002/emp2.12782

3. Bosch NA, Law AC, Rucci JM, Peterson D, Walkey AJ. Predictive Validity of the Sequential Organ Failure Assessment Score versus Claims-based Scores among Critically Ill Patients. *Ann Am Thorac Soc*. 2022;19(6):1072-1076. doi:10.1513/AnnalsATS.202111-1251RL

4. Lodise TP, Chopra T, Nathanson BH, Sulham K. Hospital admission patterns of adult patients with complicated urinary tract infections who present to the hospital by disease acuity and comorbid conditions: How many admissions are potentially avoidable? *Am J Infect Control*. 2021;49(12):1528-1534. doi:10.1016/j.ajic.2021.05.013

5. Aronsky D, Haug PJ, Lagor C, Dean NC. Accuracy of administrative data for identifying patients with pneumonia. *Am J Med Qual*. 2005;20(6):319-328. doi:10.1177/1062860605280358

6. Centers for Medicare & Medicaid Services. ICD-10 General Equivalence Mappings: An Introduction. https://www.cms.gov/Medicare/Coding/ICD10/downloads/ICD10MappingFactSheetIntroduction.pdf

7. Lodise TP, Izmailyan S, Olesky M, Lawrence K. An Evaluation of Treatment Patterns and Associated Outcomes Among Adult Hospitalized Patients With Lower-Risk Community-Acquired Complicated Intra-abdominal Infections: How Often Are Expert Guidelines Followed? *Open Forum Infect Dis*. 2020;7(7):ofaa237. doi:10.1093/ofid/ofaa237
